# Assessment of brain age in posttraumatic stress disorder: Findings from the ENIGMA PTSD and brain age working groups

**DOI:** 10.1002/brb3.2413

**Published:** 2021-12-14

**Authors:** Ashley N. Clausen, Kelene A. Fercho, Molly Monsour, Seth Disner, Lauren Salminen, Courtney C. Haswell, Emily Clarke Rubright, Amanda A. Watts, M. Nicole Buckley, Adi Maron‐Katz, Anika Sierk, Antje Manthey, Benjamin Suarez‐Jimenez, Bunmi O. Olatunji, Christopher L. Averill, David Hofmann, Dick J. Veltman, Elizabeth A. Olson, Gen Li, Gina L. Forster, Henrik Walter, Jacklynn Fitzgerald, Jean Théberge, Jeffrey S. Simons, Jessica A. Bomyea, Jessie L. Frijling, John H. Krystal, Justin T. Baker, K. Luan Phan, Kerry Ressler, Laura K. M. Han, Laura Nawijn, Lauren A. M. Lebois, Lianne Schmaal, Maria Densmore, Martha E. Shenton, Mirjam van Zuiden, Murray Stein, Negar Fani, Raluca M. Simons, Richard W. J. Neufeld, Ruth Lanius, Sanne van Rooij, Saskia B.J. Koch, Serena Bonomo, Tanja Jovanovic, Terri deRoon‐Cassini, Timothy D. Ely, Vincent A. Magnotta, Xiaofu He, Chadi G. Abdallah, Amit Etkin, Christian Schmahl, Christine Larson, Isabelle M. Rosso, Jennifer Urbano Blackford, Jennifer S. Stevens, Judith K. Daniels, Julia Herzog, Milissa L. Kaufman, Miranda Olff, Richard J. Davidson, Scott R. Sponheim, Sven C. Mueller, Thomas Straube, Xi Zhu, Yuval Neria, Lee A. Baugh, James H. Cole, Paul M. Thompson, Rajendra A. Morey

**Affiliations:** ^1^ VA Mid‐Atlantic Mental Illness Research Education, and Clinical Center Durham North Carolina USA; ^2^ Duke University Brain Imaging and Analysis Center Durham North Carolina USA; ^3^ Kansas City VA Medical Center Kansas City Missouri USA; ^4^ ARQ National Psychotrauma Centrum Diemen The Netherlands; ^5^ Brain Health Research Centre Department of Anatomy University of Otago Dunedin New Zealand; ^6^ Center for Brain and Behavior Research University of South Dakota Vermillion South Dakota USA; ^7^ Center for Healthy Minds Departments of Psychology and Psychiatry University of Wisconsin‐Madison Madison Wisconsin USA; ^8^ Centre for Medical Image Computing Computer Science University College London London UK; ^9^ Centre for Youth Mental Health The University of Melbourne Melbourne Australia; ^10^ Civil Aerospace Medical Institute US Federal Aviation Administration Oklahoma City Oklahoma USA; ^11^ Clinical Neuroscience Division National Center for PTSD West Haven Connecticut USA; ^12^ Columbia University Medical Center Manhattan New York USA; ^13^ Dementia Research Centre Institute of Neurology University College London London UK; ^14^ Department of Experimental Clinical and Health Psychology Ghent University Ghent Belgium; ^15^ Department of Medical Biophysics University of Western Ontario London Ontario Canada; ^16^ Department of Neuroscience Western University London Ontario Canada; ^17^ Department of Personality Psychological Assessment and Treatment University of Deusto Bilbao Spain; ^18^ Department of Psychiatry and Behavioral Health The Ohio State University Columbus Ohio USA; ^19^ Department of Psychiatry and Behavioral Neurosciences Wayne State University Detroit Michigan USA; ^20^ Department of Psychiatry and Behavioral Sciences Emory University School of Medicine Atlanta Georgia USA; ^21^ Department of Psychiatry and Behavioral Sciences Stanford University of Medicine Stanford California USA; ^22^ Department of Psychiatry and Behavioral Sciences Vanderbilt University Medical Center Nashville Tennessee USA; ^23^ Department of Psychiatry Amsterdam University Medical Centers Location Academic Medical Center Amsterdam Neuroscience University of Amsterdam Amsterdam The Netherlands; ^24^ Department of Psychiatry Amsterdam University Medical Centers Location VU University Medical Center VU University Amsterdam The Netherlands; ^25^ Department of Psychiatry Amsterdam University Medical Centers VU University Medical Center GGZ inGeest Amsterdam Neuroscience Amsterdam The Netherlands; ^26^ Department of Psychiatry Harvard Medical School Boston Massachusetts USA; ^27^ Department of Psychiatry University of Minnesota Medical School Minneapolis Minnesota USA; ^28^ Department of Psychiatry VA Boston Healthcare System Brockton Massachusetts USA; ^29^ Department of Psychiatry Western University London Ontario Canada; ^30^ Department of Psychiatry Yale University School of Medicine New Haven Connecticut USA; ^31^ Department of Psychology University of British Columbia Okanagan Canada; ^32^ Department of Psychology University of Chinese Academy of Sciences Beijing China; ^33^ Department of Psychology University of South Dakota Vermillion South Dakota USA; ^34^ Department of Psychology Vanderbilt University Nashville Tennessee USA; ^35^ Department of Psychosomatic Medicine and Psychotherapy Central Institute of Mental Health Medical Faculty Mannheim/Heidelberg University Mannheim Germany; ^36^ Department of Veterans Affairs Tennessee Valley Healthcare System Nashville Tennessee USA; ^37^ Departments of Psychiatry & Radiology Harvard Medical School Boston Massachusetts USA; ^38^ Departments of Radiology Psychiatry and Biomedical Engineering University of Iowa Iowa City Iowa USA; ^39^ Division of Basic Biomedical Sciences Sanford School of Medicine University of South Dakota Vermillion South Dakota USA; ^40^ Division of Depression and Anxiety Disorders McLean Hospital Belmont Massachusetts USA; ^41^ Division of Women's Mental Health McLean Hospital Belmont Massachusetts USA; ^42^ Donders Institute for Brain Cognition and Behaviour Centre for Cognitive Neuroimaging Radboud University Nijmegen Nijmegen The Netherlands; ^43^ Harvard Medical School Boston Massachusetts USA; ^44^ Imaging Division Lawson Health Research Institute London Ontario Canada; ^45^ Institute for Technology in Psychiatry McLean Hospital Harvard University Belmont Massachusetts USA; ^46^ Institute of Medical Psychology and Systems Neuroscience University of Muenster Muenster Germany; ^47^ Laboratory for Traumatic Stress Studies CAS Key Laboratory of Mental Health Institute of Psychology Chinese Academy of Sciences Beijing China; ^48^ Marquette University Milwaukee Wisconsin USA; ^49^ McLean Hospital Belmont Massachusetts USA; ^50^ Medical College of Wisconsin Milwaukee Wisconsin USA; ^51^ Minneapolis VA Health Care System Minneapolis Minnesota USA; ^52^ New York State Psychiatric Institute New York New York USA; ^53^ Orygen The National Centre of Excellence in Youth Mental Health Parkville Australia; ^54^ Psychiatry Neuroimaging Laboratory Brigham and Women's Hospital Boston Massachusetts USA; ^55^ Sioux Falls VA Health Care System Sioux Falls South Dakota USA; ^56^ UC San Diego Department of Family Medicine and Public Health San Deigo California USA; ^57^ UC San Diego Department of Psychiatry San Deigo California USA; ^58^ University Medical Centre Charite Berlin Germany; ^59^ University of Groningen The Netherlands; ^60^ University of Wisconsin‐Milwaukee Milwaukee Wisconsin USA; ^61^ VA San Diego Healthcare System Center of Excellence for Stress and Mental Health San Deigo California USA; ^62^ Wu Tsai Neuroscience Institute Stanford University Stanford California USA; ^63^ Department of Psychology Western University London Ontario Canada; ^64^ Imaging Genetics Center Mark & Mary Stevens Neuroimaging & Informatics Institute Keck School of Medicine University of Southern California Marina del Rey California USA; ^65^ Michael E DeBakey VA Medical Center Houston Texas USA; ^66^ Menninger Department of Psychiatry Baylor College of Medicine Houston Texas USA

**Keywords:** aging, machine learning, mega‐analysis, neuroimaging, posttraumatic stress disorder, trauma

## Abstract

**Background:**

Posttraumatic stress disorder (PTSD) is associated with markers of accelerated aging. Estimates of brain age, compared to chronological age, may clarify the effects of PTSD on the brain and may inform treatment approaches targeting the neurobiology of aging in the context of PTSD.

**Method:**

Adult subjects (*N* = 2229; 56.2% male) aged 18–69 years (mean = 35.6, SD = 11.0) from 21 ENIGMA‐PGC PTSD sites underwent T1‐weighted brain structural magnetic resonance imaging, and PTSD assessment (PTSD+, *n* = 884). Previously trained voxel‐wise (brainageR) and region‐of‐interest (BARACUS and PHOTON) machine learning pipelines were compared in a subset of control subjects (*n* = 386). Linear mixed effects models were conducted in the full sample (those with and without PTSD) to examine the effect of PTSD on brain predicted age difference (brain PAD; brain age − chronological age) controlling for chronological age, sex, and scan site.

**Results:**

BrainageR most accurately predicted brain age in a subset (*n* = 386) of controls (brainageR: ICC = 0.71, *R* = 0.72, MAE = 5.68; PHOTON: ICC = 0.61, *R* = 0.62, MAE = 6.37; BARACUS: ICC = 0.47, *R* = 0.64, MAE = 8.80). Using brainageR, a three‐way interaction revealed that young males with PTSD exhibited higher brain PAD relative to male controls in young and old age groups; old males with PTSD exhibited lower brain PAD compared to male controls of all ages.

**Discussion:**

Differential impact of PTSD on brain PAD in younger versus older males may indicate a critical window when PTSD impacts brain aging, followed by age‐related brain changes that are consonant with individuals without PTSD. Future longitudinal research is warranted to understand how PTSD impacts brain aging across the lifespan.

## Graphical abstract

Posttraumatic stress disorder (PTSD) is associated with markers of accelerated aging. We explored estimates of brain age, as compared to chronological age, as a possible marker of accelerated aging in PTSD populations leveraging a large multi‐site sample. We identified an interaction of PTSD, age, and sex which showed young males with PTSD had a greater brain predicted age difference [PAD] than young male controls, young females with PTSD, and young females without PTSD. A similar pattern was present in middle‐aged males, but the effect of PTSD was weaker than in young adults. Male controls in the old subgroup exhibited higher brain‐PAD than old males with PTSD, old females with PTSD, and old females without PTSD.

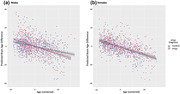

## 1 INTRODUCTION

Exposure to stress plays an important role in the development of medical conditions (Cohen & Manuck, 1995). A prime example is the relationship between posttraumatic stress disorder (PTSD) and increased risk for developing cardiovascular (Edmondson & von Känel, 2017) and cardiometabolic (Edmondson & von Känel, 2017) disease. Critically, PTSD has been linked with increased mortality (Edmondson & von Känel, 2017), underscoring the relationships between mental and physical health. A number of behavioral and biological mediators have been examined including sleep dysregulation, cigarette use, decreased physical activity, autonomic reactivity, and inflammation, which may all contribute to accelerated cellular aging and subsequently increased rates of medical morbidity (Wolf & Schnurr, 2016). Identification of accelerated aging processes may aid in detecting elevated risk for developing disease and provide opportunities for a personalized medicine approach to treatment.

Specific to PTSD, biomarkers of accelerated aging have been identified in the epigenome, and immune and inflammatory systems (Wolf & Morrison, 2017). Telomere length and DNA “methylation age” are two biomarkers that have shown mixed results (Wolf & Morrison, 2017), and may be more or less sensitive depending on the type of outcome measure (e.g., current vs. lifetime PTSD or specific symptom clusters). In a recent meta‐analysis, lifetime PTSD symptom severity and childhood trauma were associated with accelerated DNA methylation age (Katrinli et al., 2020; Wolf et al., 2018; Yang et al., 2020). PTSD has also been linked with diminished immune response (Aiello et al., 2016), and a heightened inflammatory response based on C‐reactive protein (Spitzer et al., 2010), TNF‐alpha (von Kanel et al., 2007), IL‐6, and IL‐1β (Newton et al., 2014), which may impact aging processes. While peripheral biomarkers provide initial evidence for a link between PTSD and accelerated aging, identification of neural biomarkers is critical to understanding the complex relationships between PTSD, aging, and the brain. Given the known deleterious effects of aging on the brain (Schmitz et al., 2018), coupled with functional and structural brain changes associated with PTSD (Clausen et al., 2020; Logue et al., 2018; Morey et al., 2020), neural markers of accelerated aging in PTSD may help to stratify disease risk, clarify the pathology of psychiatric disease on the brain, and aid in the development of tailored treatments for PTSD.

Machine learning algorithms have been applied to metrics derived from T1‐weighted structural MRI to predict biological brain age (Cole & Franke, 2017), which can be compared with chronological age to calculate a difference score, referred to as brain predicted age difference (PAD). Brain PAD has been optimized by training algorithms using chronological age in large samples of healthy individuals (Cole & Franke, 2017) and has been tested in aging (J. H. Cole et al., 2018), schizophrenia, bipolar disorder, and depression (Besteher et al., 2019; Han et al., 2021; Schnack et al., 2016). In aging, higher brain PAD, which is indicative of an "older appearing" brain, has been linked with weaker grip strength, diminished lung function, slower walking speed, lower fluid intelligence, higher allostatic load, and critically, increased mortality risk (Cole et al., 2018). Patients with schizophrenia and bipolar disorder exhibit, on average, higher brain PAD, suggesting that these disorders may be associated with accelerated brain aging (Hajek et al., 2019; Schnack et al., 2016). By contrast, findings with brain PAD in patients with depression have been inconsistent (Besteher et al., 2019; Han et al., 2021), but suggest a small effect of depression on brain PAD. Research examining brain PAD in PTSD is in its infancy, but initial evidence suggests PTSD is associated with accelerated brain aging. In a relatively large sample (*N* = 804) of individuals aged 8–21, subjects with PTSD exhibited higher brain PAD relative to controls (Liang et al., 2019). However, the study was conducted in children, adolescents, and very young adults, and group sample sizes were highly imbalanced (PTSD, *n* = 70; control, *n* = 734); the control group also lacked trauma exposure.

The primary aim of the present study was to examine the relationship between PTSD and brain PAD in adults with and without PTSD who were aggregated from 21 sites in the ENIGMA‐PTSD Consortium. As PTSD has been linked with other markers of accelerated aging (Li et al., 2017; Wolf et al., 2018), we hypothesized that the PTSD group would show, on average, higher brain PAD. A wide range of different brain age prediction techniques are available (Cole et al., 2019). Therefore, prior to embarking on our primary goal of investigating brain aging in PTSD, a test set of controls was used to evaluate the performance of three machine learning‐based pipelines including one voxel‐based algorithm and two algorithms that rely on region‐of‐interest (ROI) cortical and subcortical measures from the FreeSurfer 5.3 segmentation. We hypothesized the voxel‐based method, which uses greater structural detail than the ROI approaches, would provide more accurate estimates of brain age.

## 2 METHOD

### 2.1 Subjects

Subjects included 2315 adults from 21 sites participating in the ENIGMA‐PGC PTSD Consortium (see Table 1 for demographic and clinical characteristics). Each site had unique inclusion and exclusion criteria. Common exclusion criteria included meeting current diagnostic criteria for a substance use disorder, neurological disorder, active psychosis, and moderate‐to‐severe head injuries. Subjects were included in the present analysis if they completed a 3D T1‐weighted brain scan with magnetic resonance imaging (MRI), and assessment of PTSD, as well as reported age and sex. Eighty‐six subjects were removed from the analysis due to missing data (see Supporting Information for subject exclusion). Therefore, a total of 2229 subjects with (*n* = 882) and without (*n* = 1347) PTSD were included in the final analysis. Control subjects included trauma‐exposed (*n* = 1226) and unexposed (*n* = 118) individuals. Three control subjects were missing trauma‐exposure data. Analyses were conducted with and without unexposed controls and those missing trauma exposure data. No significant differences were identified in the main or supplemental analyses; thus, unexposed controls, and those missing trauma‐exposure data, were included in the analyses.

**TABLE 1:** Demographic characteristics

	Full group (*N* = 2229)	Controls (*n* = 1347)	PTSD (*n* = 884)	Comparison between groups
	**Mean (SD)**	**Mean (SD)**	**Mean (SD)**	***t*‐value (*p*‐value)**
Age	35.6 (11.0)	35.4 (11.0)	35.9 (10.9)	−0.90 (.370)
Predicted age	35.1 (10.4)	34.7 (10.6)	35.6 (10.1)	−1.91 (.056)
Brain PAD	−0.5 (8.7)	−0.7 (8.4)	−0.3 (9.0)	−1.17 (.242)
Childhood trauma severity	*N* = 1352, 1.3 (0.86)	*n* = 846, 1.1 (0.9)	*n* = 506, 1.6 (0.7)	−12.44 (< .001)
	**% with (*n*)**	**% with (*n*)**	**% with (*n*)**	**X^2^ or F‐statistic (*p*‐value)**
Males	56.2% (1252)	57.8% (778)	53.6% (474)	3.33 (.068)
% Caucasian	54.6% (1217)	55.8% (752)	52.6% (465)	14.61 (.001)
MDD diagnosis	*N* = 1878, 32.6% (613)	*n* = 1142, 11.8% (135)	*n* = 736, 64.9% (478)	572.07 (<.001)
Military status	*N* = 2102, 42.1% (884)	*n* = 1238, 45.7% (566)	*n* = 864, 36.8% (318)	16.23 (<.001)

*Abbreviations*: PAD, predicted age difference; MDD, major depressive disorder; PTSD, posttraumatic stress disorder; SD, standard deviation.

### 2.2 Clinical assessment

A breakdown of clinical assessments by site is included in Tables S1 and S2. Clinical assessments were conducted for PTSD, depression, and childhood trauma using either self‐report measures or clinician‐administered interviews. When available, severity scores were used to estimate PTSD and depression diagnosis using previously established cut‐off scores (Table S1).

#### 2.2.1 PTSD

Current PTSD diagnosis was assessed with one of the following instruments: Clinician Administered PTSD Scale for DSM‐IV (Pfohl et al., 1997) or DSM‐5 (Weathers et al., 2018), the PTSD Symptom Checklist for DSM‐IV Civilian (Weathers et al., 2015), Military (Yarvis et al., 2012) versions, or for DSM‐5 (Weathers et al., 2015), Davidson Trauma Scale (Davidson, 1996), the structured clinical interview for DSM‐IV (Spitzer et al., 1992) or DSM‐5 (First et al., 2014) disorders (SCID), or the Mini International Neuropsychiatric Inventory (MINI) 6.0 (Sheehan & Lecrubier, 2010) or 7.0 (Sheehan et al., 2014).

#### 2.2.2 Childhood trauma

Thirteen of the 21 participating sites collected information on childhood trauma. The majority of sites (*n* = 11) used the Childhood Trauma Questionnaire (Bernstein et al., 2003). One site used the Early Trauma Inventory (Bremner et al., 2000), and one site used the Stressful Life Events Screening Questionnaire—Revised (Green et al., 2006). Childhood trauma measures were harmonized to yield a score of 0 (no trauma exposure), 1 (exposure to one type [e.g., physical vs. emotional abuse] of trauma), or 2 (exposure to two or more types of traumas).

#### 2.2.3 Depression

Twenty of the 21 participating sites diagnosed depression and/or its severity with one of the following instruments: the Beck Depression Inventory–II (Beck et al., 1996) and 1A (Steer et al., 1999), the Hamilton Rating Scale for Depression (Bremner et al., 2000), the Hospital Anxiety and Depression Scale (Snaith & Zigmond, 2000), the Physical Health Questionnaire−9 (Kroenke & Spitzer, 2002), the Center for Epidemiology Studies – Depression (Orme et al., 1986), the Depression and Anxiety Stress Scale – depression subscale (Lovibond & Lovibond, 1995), the MINI, or the SCID.

### 2.3 MRI acquisition and preprocessing

A high resolution T1‐weighted brain MRI scan, optimized for tissue contrast, was acquired at each site and was analyzed locally (Table S2). Raw T1‐weighted images were then sent to Duke University for preprocessing and quality assurance using standardized ENIGMA protocols to harmonize image analyses across multiple sites. Raw and preprocessed images (for FreeSurfer 5.3) were visually inspected for pathology, image quality, and quality of automated segmentation (http://enigma.ini.usc.edu/protocols/imaging‐protocols/). All scans were preprocessed using the same version of FreeSurfer to minimize the variability between segmentation results. Version 5.3 was chosen based on previously established preprocessing procedures for a previously trained brain age algorithm.

### 2.4 Machine learning pipelines

We examined three pre‐trained machine learning pipelines to estimate brain age in a subset of controls (*n* = 386). One algorithm used voxel‐based data, referred to as brainageR and two algorithms, PHOTON Brain Age (PHOTON‐BA; https://www.photon‐ai.com/enigma_brainage) and Brain‐Age Regression Analysis and Computation Utility Software (BARACUS), and used cortical and subcortical segmentation results from FreeSurfer 5.3. Descriptions of each model are included in the Supporting Information. Briefly, brainageR applies a Gaussian process regression model to T1‐weighted scans, parcellating gray and white matter, to predict chronological age (Cole et al., 2018) (https://github.com/james‐cole/brainageR; https://doi.org/10.5281/zenodo.3476365 ). While both PHOTON‐BA and BARACUS rely on FreeSurfer segmented data, PHOTON‐BA estimates brain age by applying a ridge regression model (Schmaal et al., 2020) whereas BARACUS uses a linear support vector regression model (Liem et al., 2017).

### 2.5 Statistical analysis

All analyses were conducted in R Statistical Software Package (freely available at https://cran.r‐project.org). To assess model performance, we calculated intraclass correlations (ICC) between chronological and predicted age, proportion of the variance explained by the model (*R* ^2^), Pearson's *R* between predicted brain age and chronological age, mean absolute error (MAE), and root mean square error (RMSE) for each machine learning algorithm in a subset of control subjects. This subset was a convenience sample from three participating sites which had previously processed MRI data and calculated estimates for brain age using the three machine learning models (Minneapolis VA, Duke University, University of South Dakota). The algorithm with the highest *R*, *R* ^2^, and ICC values, and lowest RMSE and MAE for control subjects, was applied to the full sample from 21 participating sites to examine relationships between brain age estimates and PTSD.

Brain PAD (predicted brain age − chronological age) was calculated for each subject, using the algorithm with the best fit, and was used as the primary outcome measure. Brain PAD is typically overestimated for children and young adults and underestimated in older individuals, which is explained by the well‐known statistical phenomenon of regression to the mean (Le et al., 2018; Liang et al., 2019). Two methods have been proposed to adjust for this phenomenon, including incorporating age as a covariate in the main analysis (Le et al., 2018; Liang et al., 2019) and developing residualized brain PAD scores by regressing chronological age onto brain PAD (Le et al., 2018). Because we aimed to examine main and interaction effects of age, we incorporated chronological age as a covariate, as opposed to developing a residualized brain PAD score. To ensure age did not significantly differ between groups with and without PTSD, we examined differences in age between control and PTSD groups. To mega‐analyze differences in brain PAD between subjects with and without PTSD, we used linear mixed effects (LME) modeling (R Library *lme4*; R command *lmer*). PTSD diagnosis, age, age^2^, and sex were then entered into the LME as fixed effects. Age and age^2^ were centered prior to performing the analysis. As subjects were nested within each site, the site was included as a random effect in all models to adjust for differences in scanners and systematic differences between sites. We explored main and interaction effects of all covariates and PTSD diagnosis.

*Model 1 Main Effects: brain PAD ∼ age + age^2^ + sex + PTSD diagnosis + (1 | site)*
Model 2 Interaction Effects: brain PAD ∼ age * age^2^ * sex * PTSD diagnosis + (1 | site)

We conducted supplemental analyses to explore the effects of race, childhood trauma, MDD diagnosis, and military status by adding covariates that related to brain PAD at or below the trend level (*p *< .10) to LME Model 2 as a fixed main effect. In a subsample of subjects with PTSD who completed the CAPS‐IV (*n* = 515), LME modeling analogous to the main analysis was conducted to explore effects of PTSD severity in relation to brain PAD. Findings were considered significant with a critical *p*‐value < .05. AIC and BIC indices were used to assess model fit.

Model 3 Additive Effects: brain PAD ∼ age * age^2^ * sex * PTSD diagnosis + ***covariate*** *+ (1 | site)*

## 3 RESULTS

### 3.1 Brain age prediction performance

**FIGURE 1:**
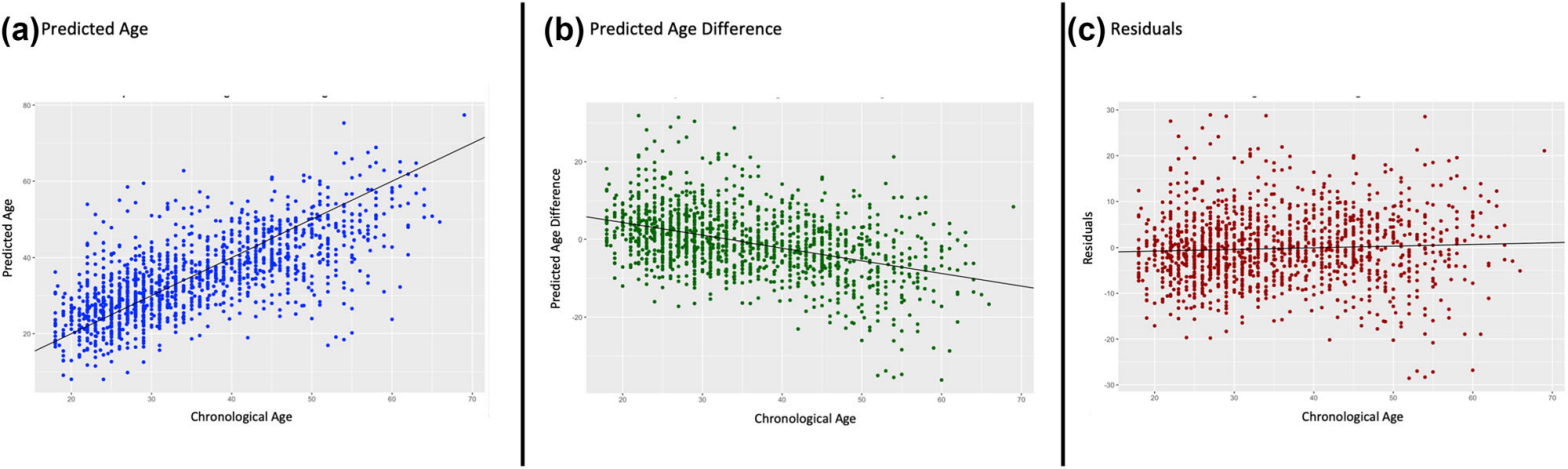
Model validation in control subjects. (a) A significant, positive relationship was identified between chronological age and predicated age in control subjects (*R* = 0.70, *R* ^2^ = 0.49, *p *> .0001). (b) A significant negative relationship was observed between chronological age and predicated age difference (predicted age − chronological age) in control subjects (*R* = −0.33, *R* ^2^ = 0.18, *p* > .0001). (c) While the effect was small, we continued to observe a significant relationship when plotting the residual effects of the linear relationship between predicted age difference and chronological age (shown in Figure 1b) with chronological age in controls (*R* = 0.04, *R* ^2^ = 0.002, *p *= .05)

We examined the predictive ability of the brainageR, PHOTON‐BA, and BARACUS machine learning pipelines to estimate brain age in a subsample of control subjects (*n* = 386) from three sites. Results of pipeline comparisons are presented in the Supporting Information and Figures S1–S3. Briefly, brainageR demonstrated a stronger relationship between chronological and predicted age (*R* = 0.72) and lower error (MAE = 5.68) compared to BARACUS (*R* = 0.64; MAE = 8.8) and PHOTON‐BA (*R* = 0.62; MAE = 6.38) models. Model validation for the brainageR pipeline is displayed in Figure 1.

### 3.2 Covariates

**FIGURE 2:**
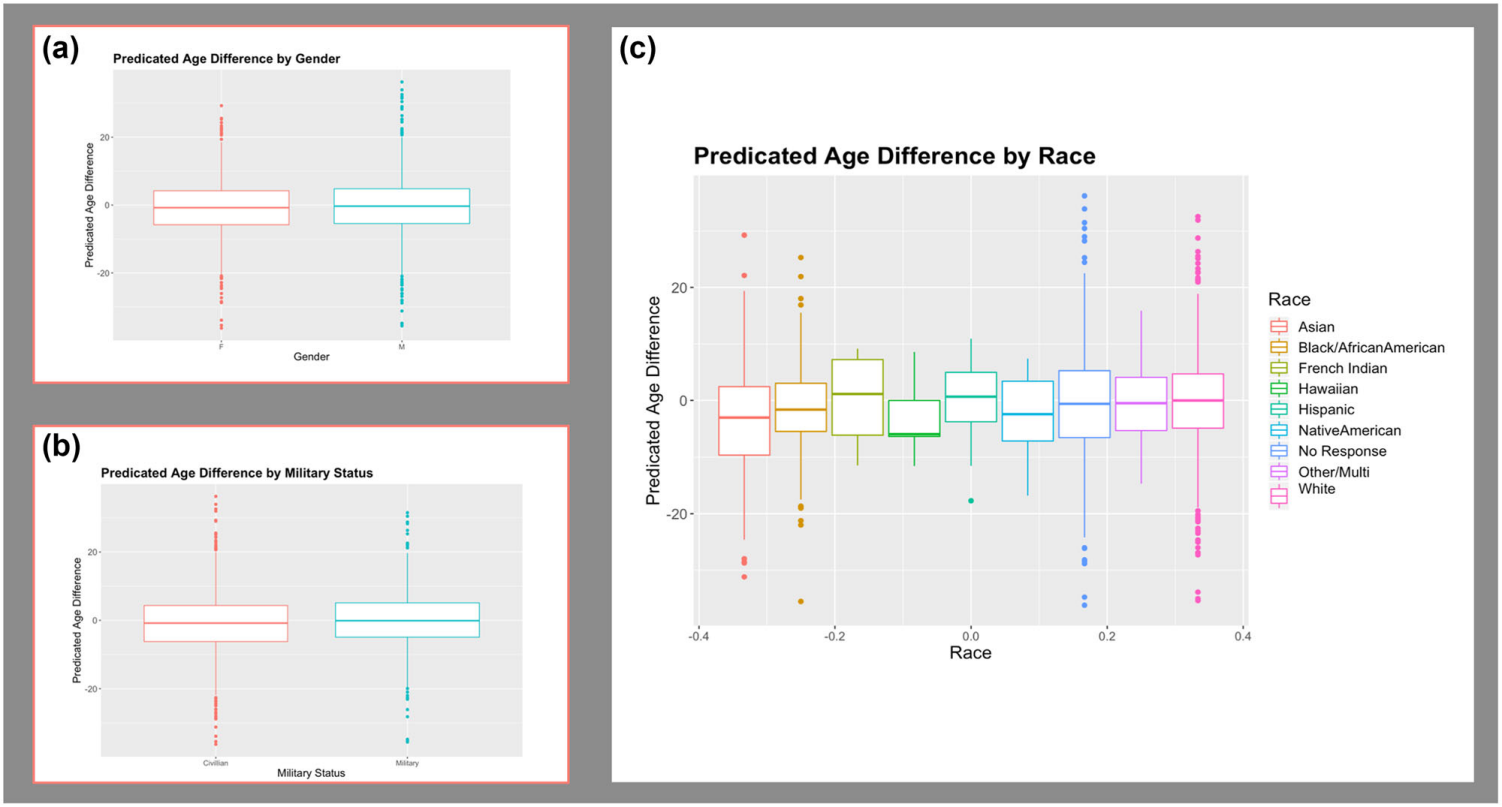
Potential covariates. (a) Predicted age difference by sex. (b) Predicted age difference by military status. (c) Predicted age difference by each racial or ethnic group (driven solely by differences in the Beijing data). Sex was included as a covariate in the main analysis. Both military status and race were examined in supplementary analyses

**FIGURE 3:**
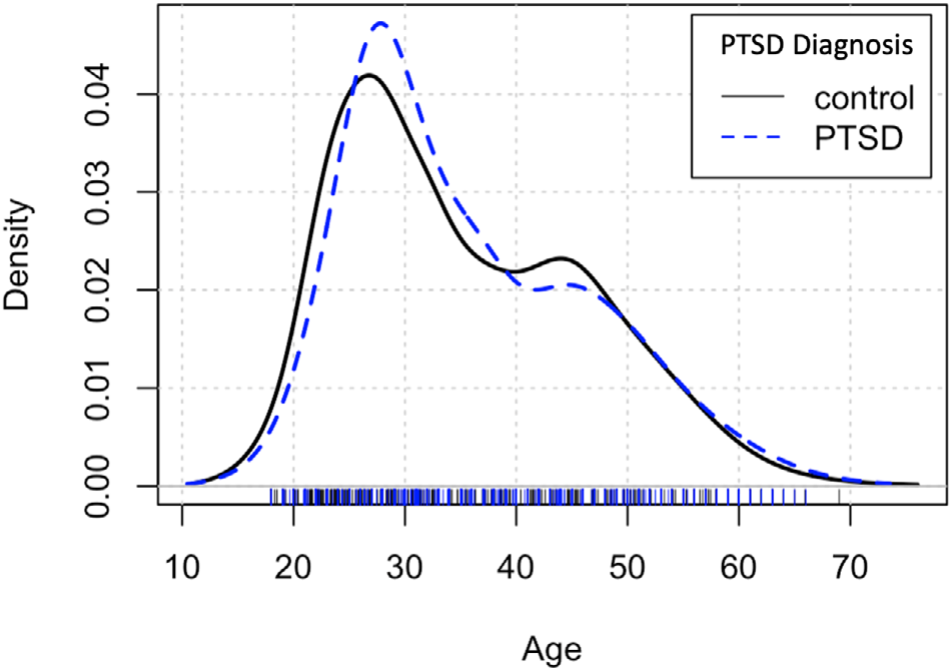
Age density for diagnostic groups. The probability density as a function of subjects’ age in the PTSD and control groups

For the main analysis, we examined linear and nonlinear effects of age, and sex, with brainageR brain PAD. Age (r(2227) = −0.45, *p *< .001), age^2^ (r(2227) = −0.45, *p *< .001), and sex (t(2227) = −1.84, *p *= .06), when examined separately, were related (*p *< .1) to brain PAD. For the supplementary analyses, we examined other potentially contributing factors including race, military status, depression, and childhood trauma. Both race (F[1,2227] = 4.768, *p *= .029), and military status (t(2104) = −3.0858, *p *= .002) when examined separately, significantly related to brain PAD. Neither childhood trauma (F(1,1350) = 1.78, *p *= .182) nor depression (t(1876) = −0.99, *p *= .321) related to brain PAD. See Figure 2 for visualization of covariates. We also examined the relationship between age and PTSD diagnosis. Age did not significantly differ between groups (t(2227) = −0.897, *p *= .370), and there was a high degree of overlap across distributions in age for the PTSD and control groups (Figure 3).

### 3.3 Impact of PTSD diagnosis on brain age

Results for each LME model are presented in Table 2. Model 1 examined the main effects of age, age^2^, sex, and PTSD on brain PAD. Age (*β* = −0.38, *p *< .001) and sex (*β* = 0.84, *p* = .027) significantly related to brain PAD. There was no main effect of age^2^ (*β* < 0.001, *p *= .520) or PTSD diagnosis (*β* = −0.280, *p *= .420).

**TABLE 2:** Linear mixed effects (LME) results for PTSD diagnosis

		Beta	CI	*t*‐value	*p*
***Model 1*** *AIC = 15,344.3, BIC = 15,384.3, N = 2229, Marginal R^2^ / Conditional R^2^ = 0.220 / 0.266*					
	Intercept	—1.13	−2.18 to −0.08	−2.12	.040
	PTSD	0.28	−0.40 – 0.96	0.81	.420
	Age	−0.38	−0.41 to −0.34	−22.38	<.001*
	Age^2^	0.00	−0.00 – 0.00	0.644	.520
	Sex (M)	0.84	0.10 – 1.58	2.21	.027*
** ***Model 2*** *AIC = 15,336.9, BIC = 15,399.7, N = 2229, Marginal R^2^ / Conditional R^2^ = 0.226 / 0.270*					
	Intercept	−1.36	−2.45 to −0.28	−2.46	.017
	PTSD	0.75	−0.29 – 1.80	1.43	.153
	Age	−0.40	−0.45 to −0.34	−13.94	<.001*
	Age^2^	0−0	−0.00 – 0.00	0.69	.493
	Sex (M)	1.15	0.26 – 2.04	2.53	.017*
	PTSD*Age	−0.00	−0.09 – 0.08	−0.14	.890
	PTSD*Sex (M)	−0.76	−2.09 – 0.57	−1.11	.265
	Age*Sex (M)	0.09	0.02 – 0.16	2.39	.017*
	PTSD*Age*Sex (M)	−0.14	−0.26 to −0.02	−2.34	.019*
***Model 3*** *AIC = 15,338.7, BIC = 15,407.3, N = 2229, Marginal R^2^ / Conditional R^2^ = 0.227 / 0.270*					
	Intercept	−1.23	−2.46 to −0.01	−1.97	.053
	PTSD	0.76	−0.28 – 1.80	1.43	.150
	Age	−0.40	−0.45 to −0.34	−13.93	<.001*
	Age^2^	0.00	−0.00 – 0.00	0.69	.488
	Sex (M)	1.14	0.25 – 2.04	2.50	.012*
	Race	−0.03	−0.18 – 0.11	−0.44	.664
	PTSD*Age	−0.01	−0.09 – 0.08	−0.15	.883
	PTSD*Sex (M)	−0.76	−2.09 – 0.58	−1.11	.267
	Age*Sex (M)	0.09	0.02 – 0.16	2.38	.018*
	PTSD*Age*Sex (M)	−0.14	−0.26 to −0.02	−2.33	.020*
***Model 4*** *AIC = 14,472.6, BIC = 14,540.4, N = 2102, Marginal R^2^ / Conditional R^2^ = 0.247 / 0.292*					
	Intercept	–1.75	−2.93 to −0.57	−2.91	.005
	PTSD	0.79	−0.26 – 1.85	1.48	.140
	Age	−0.42	−0.48 to −0.37	−14.00	<.001*
	Age^2^	0.00	−0.00 – 0.00	0.64	.524
	Sex (M)	1.11	0.14 – 2.08	2.25	.025
	Military status	1.06	−0.25 – 2.36	1.59	.114
	PTSD*Age	0.02	−0.07 – 0.10	0.40	.689
	PTSD*Sex (M)	−0.88	−2.25 – 0.48	−1.27	.206
	Age*Sex (M)	0.09	0.01 – 0.16	2.17	.030
	PTSD*Age*Sex (M)	−0.15	−0.27 to −0.03	−2.4	.017

*Abbreviations*: LME, linear mixed effects; PTSD, posttraumatic stress disorder; AIC, Akaike's information criteria; BIC, Bayesian information criteria.

** Indicates best model fit.

**FIGURE 4:**
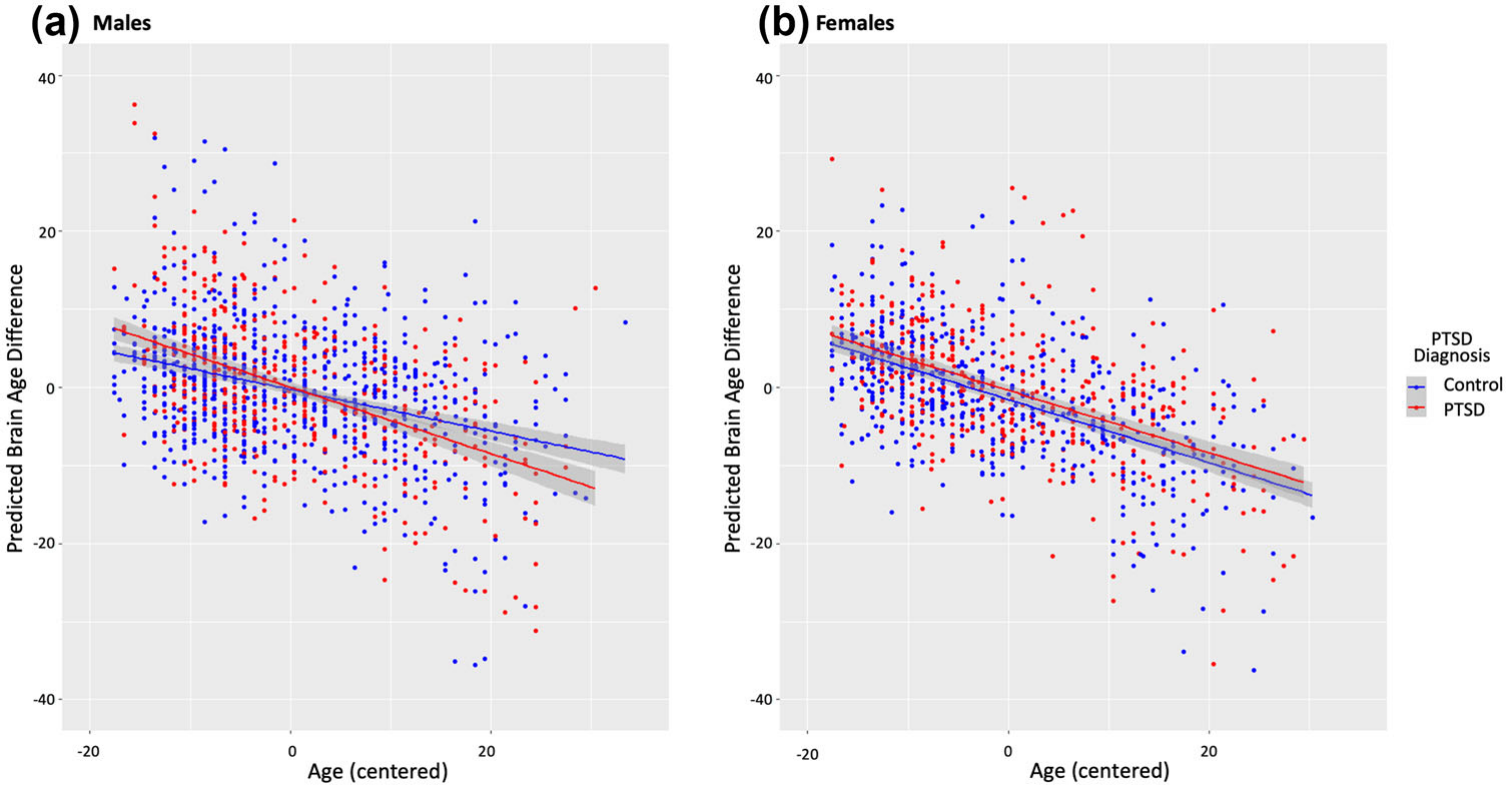
Relationships between PTSD, chronological age, age^2^ and sex with predicted brain age difference (PAD). Brain PAD scores were calculated using the brainageR pipeline. Main and interaction effects of PTSD, age (centered), and sex with main fixed effects of age^2^ (centered) and site included as a random effect are displayed. (a) Linear plot of age by PTSD diagnosis interaction in male subjects. Males with PTSD exhibited a steeper, negative relationship between age and brain PAD relative to males without PTSD. (b) Linear plot of age by PTSD diagnosis in female subjects. No interaction was observed between PTSD diagnosis and age in female subjects. Females with PTSD exhibited qualitatively higher brain PAD relative to females without PTSD

Next, we explored interaction effects of age, sex, and PTSD diagnosis on brain PAD (Model 2, Figure 4). We identified a three‐way interaction between age, sex, and PTSD diagnosis (*β* = −0.14, *p *= .019). This model exhibited lower AIC, but higher BIC relative to Model 1 Revised (Model 1: AIC = 15,344.3, BIC = 15,384.3; Model 2: AIC = 15,336.9, BIC = 15,399.7). BIC penalizes model complexity more heavily than AIC. Thus, it is expected that the interaction model has a higher BIC relative to examination of only main effects. Model 2 was selected as the model of best fit.

To visually characterize the three‐way interaction, we divided the sample by sex (Figure 4) and plotted the relationship between age and predicted brain age by PTSD status. Males with PTSD exhibited a steeper relationship between chronological age and brain PAD compared to males without PTSD. Males with PTSD exhibited higher brain PAD at a younger age, and lower brain PAD at an older age compared to males without PTSD. Whereas females with PTSD tended to have higher brain PAD relative to females without PTSD with the exception of younger control subjects and old subjects with PTSD.

In order to further explore the three‐way interaction, we conducted pairwise comparisons of the relationship between chronological age and brain PAD in males and females with and without PTSD using the package cocor in R (Diedenhofen & Musch, 2015). Females with (Fischer's *z* = 3.54, *p* < .001) and without PTSD (Fischer's *z* = 5.15, *p* < .001) exhibited a stronger negative correlation between chronological age and brain PAD relative to males without PTSD. No differences were observed in the relationship of chronological age and brain PAD between males with and without PTSD (Fischer's *z* = 1.43, *p* = .154), females without PTSD and males with PTSD (Fischer's *z* = −1.79, *p* = .074), females with and without PTSD (Fischer's *z* = −1.04, *p* = .300), nor males and females with PTSD (Fischer's *z* = 0.65, *p* = .517).

Next, we conducted supplementary analyses to examine the potential effects of race and military status. Race and military status were added as fixed effects to Model 2 separately. When added as a fixed effect to Model 2, race did not significantly contribute to brain PAD (Model 3; race *β* = −0.03, *p *= .664). Compared to Model 2 AIC and BIC, the inclusion of race as a main effect resulted in higher AIC and BIC (Model 3 AIC = 15,338.7, BIC = 15,407.3). Classification of military status was available for 2102 subjects. When added to Model 2, there was no main effect of military status (Model 4; military status *β* = 1.06, *p *= .114) on brain PAD. As Model 4 was completed in a subset of subjects, we are unable to directly compare AIC and BIC to previous models.

### 3.4 Influence of PTSD symptom severity on brain age

We repeated the above analyses in a subsample of subjects with a PTSD diagnosis based on the CAPS‐IV (*N* = 515) to explore PTSD symptom severity. We chose to restrict our analyses to subjects with a diagnosis of PTSD because PTSD severity was not consistently assessed across sites in trauma‐exposed controls. We used the CAPS‐IV as it was used most widely across sites to assess PTSD severity. Age, age^2^, sex, depression, childhood trauma, race, and military status were tested as covariates in this subsample.

Age (R(515) = −0.50, *p *< .001), and age^2^ (R(515) = −0.49, *p *< .001) were significantly related to brain PAD in these PTSD subjects. Sex (t(513) = −1.10, *p *= .270), depression (t(374) = −0.09, *p *= .925), childhood trauma (F(1,267) = 0.25, *p *= .612), race (F(1,513) = 0.09, *p *= .765) and military status (t(513) = −0.69, *p *= .490) were not related to brain PAD. Age, age^2^, and PTSD severity were entered as fixed effects. Site was again included as a random effect.

Age significantly predicted brain PAD (*β* = −0.47, *p *< .001); however, neither PTSD severity (*β* = −0.01, *p *= .504) nor age^2^ (*β* = 0.004, *p *= .183) was significantly related to brain PAD. The interaction between age and PTSD was also not significant (*β* < 0.001, *p *= .125). See Table S3 for PTSD severity LME model results.

## 4 DISCUSSION

In this study, we investigated the difference between chronological age and MRI‐predicted brain age. The role of other potentially relevant contributors to brain age such as sex, race, childhood trauma exposure, military service, depression, and chronological age were also investigated. To apply the most accurate method for estimating brain age from structural MRI, we first compared three leading machine learning models. We determined that the voxel‐based machine learning method based on Gaussian Process Regression (brainageR) (Cole et al., 2018) outperformed two competing ROI methods (PHOTON‐BA and BARACUS) that rely on regional mean cortical thickness, surface area, and volume generated by FreeSurfer automated segmentation. Leveraging a large, multi‐site mega‐analysis of 2229 PTSD cases and controls, we identified that brain PAD was negatively related with chronological age in control subjects, such that increasing age significantly related to lower brain PAD. Furthermore, an interaction between sex and age demonstrated females had a stronger negative correlation between brain PAD and chronological age than males. In other words, males showed greater brain aging than females and this sex difference increased with age (Figure 4). We also found an interaction of PTSD, age, and sex which showed young males with PTSD had a greater brain PAD than young male controls, young females with PTSD, and young females without PTSD. A similar pattern was present in middle‐aged males, but the effect of PTSD was weaker than in the younger subgroup. Male controls in the older subgroup exhibited higher brain PAD than older males with PTSD, older females with PTSD, and old females without PTSD.

Surprisingly, brain PAD was higher in younger subjects with PTSD than in older subjects with PTSD. Some possible interpretations are that brain age in the context of PTSD may vary across lifespan and on the basis of sex and thus may not reflect a consistent biomarker of aging in PTSD such that the younger brain is more vulnerable to aging effects of PTSD (i.e., representing a critical window for PTSD's impact on brain age) or there may be additional factors such as cardiovascular or cerebrovascular disease that may influence the relationship between age and brain PAD particularly in older individuals. It is possible the brain aging process undergoes some remediation in the chronic stage of PTSD developing greater resilience over time. This assumes that onset of PTSD in the present sample generally occurred during early or early‐mid adulthood as reported in the National Comorbidity Survey Replication, which found 23 years to be the median age of onset (Kessler et al., 2005). However, such a hypothesis can only be tested with longitudinal assessments initiated soon after initial trauma exposure. Indeed, symptoms in a subset of patients with PTSD tend to remit as the trauma becomes more distant in time (Magruder et al., 2016; Sun et al., 2018). This trajectory is consistent with the natural history of the posttraumatic period where most individuals experience acute symptoms in the hours and days following exposure to a traumatic event (Cahill & Pontoski, 2005). This acute stress response generally resolves, but may persist for more than 1 month in a smaller or larger subset of individuals, depending on the nature of the trauma; this subset is deemed to have PTSD (Kolassa et al., 2010). The relationship of brain PAD to chronological age in our results may be a methodological limitation of a one‐size‐fits‐all approach we took that may bias brain age prediction in either the youngest or oldest subgroups (Elliott et al., 2019). Other approaches may include use of classification as opposed to regression to approximate ordinal effects, or use of auxiliary loss functions in deep neural networks. An alternate, perhaps unlikely interpretation is that the usual neurobiological processes that operate in senescence are mitigated by PTSD, but this hypothesis lacks empirical support from epigenetic and inflammatory research (Katrinli et al., 2020; Miller et al., 2018). Unfortunately, there is a lack of published evidence on brain aging across the lifespan in PTSD, particularly studies on neurobiological mechanisms of action. Thus, meaningful interpretation of the present results is rather challenging without comparisons to empirical evidence.

In previous research, PTSD has been associated with higher brain PAD in relatively small sample of PTSD patients (*n* = 70) who exhibited higher brain PAD relative to control subjects (Liang et al., 2019). Relatedly, “negative fateful life events” such as death in the family or financial hardship were associated with higher predicted brain age relative to chronological age in Vietnam era twin veterans, but unfortunately associations with PTSD were not reported (Hatton et al., 2018). Consistent with the present results, higher brain PAD diminished with advancing chronological age, which was attributed to negative fateful life events being more commonplace in the early‐mid stage of life or before. Importantly, the former study was conducted in a PTSD sample that was mostly female (71%) and focused on children and young adults (ages 8–21), and the latter study was conducted in all older male adults (mean age = 62 years). The present analysis included both males (56%) and females (44%) ranging in age from 18 to 69 and thus demonstrates the differential relationship of age and brain age across age groups and sex. The present results also align with reports in schizophrenia and bipolar disorder that are characterized by a so‐called “early‐hit non‐progressive” phenomenon, where accelerated brain aging occurs at younger ages followed by age‐related brain changes that are consonant with unaffected individuals (Shahab et al., 2019).

PTSD and trauma impart prolonged stress which is associated with neurodegenerative cellular processes as a result of altered immune system gene expression, elevated gene expression of inflammatory mediators, diminished expression of antiviral processes, and synthesis of antibodies (Cole, 2014; Miller & Sadeh, 2014). The accumulation of damage to DNA within neurons triggers repair processes to be activated. However, impaired repair processes cascade to accumulation of DNA damage that produces cellular senescence, apoptosis, neurodegeneration, and premature brain aging (Coppedè & Migliore, 2010). The prolonged burden of lifetime stress has been shown to accelerate epigenetic aging via glucocorticoid signaling (Gassen et al., 2017). The foregoing evidence provides some possibilities for mechanisms operating in PTSD at the molecular level to impact accelerated brain aging, particularly in young males.

The methodology for predicting brain age from neuroimaging is relatively new and represents a nonspecific biomarker of brain development and brain health (Franke & Gaser, 2019). It captures information about a large number of variables from many brain locations (or regions) into a single composite metric. Thus, patients in the clinical setting may find this to be a readily accessible and interpretable indicator of brain health, which is adjusted for chronological age. It is conceivable that brain PAD screening could facilitate clinical trial recruitment. Subjects could be stratified by brain PAD for investigating various clinical phenomenon such as mild cognitive impairment (Liem et al., 2017), elevated risk for psychosis (Gaser et al., 2013; Koutsouleris et al., 2014), progression to Alzheimer disease, borderline personality disorder (Han et al., 2021; Koutsouleris et al., 2014), major depression, bipolar disorder (Shahab et al., 2019), schizophrenia (Koutsouleris et al., 2014; Shahab et al., 2019), and brain development more generally (Brown et al., 2012; Dosenbach et al., 2010; Smyser et al., 2016). Many of the strengths of brain PAD as a biomarker also pose significant shortcomings, which include the lack of direct information about the most predictive features of brain age, which could differ in each person. In principle, direct information about the relative importance of features used in making predictions can be extracted from most machine learning algorithms (Domingos, 2015). Knowledge of these critical features may be of little interest in some machine learning applications such as face recognition, whereas such a knowledge map is vital in neuroscience and research applications more broadly. A map of the most predictive brain features may guide future research on causal mechanisms of brain aging, but was outside the scope of the present study. Future studies with deep clinical phenotyping and longitudinal assessments of mental and somatic health—along with comorbidities, illness duration, inflammatory markers, health behaviors, and multi‐omics panels (e.g., genomics) (Amoroso et al., 2019)—will help further refine and evaluate the value of brain PAD estimates, or be used in conjunction with brain PAD estimates (Han et al., 2021). Recent application of deep learning methods such as convolutional neural networks hold the promise of enhanced performance (*R* ^2 ^= 0.87) and lower MAE (Cole et al., 2017; Jónsson et al., 2019) while also offering more specificity about the most predictive features (Cole et al., 2017).

### 4.1 Limitations and conclusion

A key strength of our study is the large sample size that allowed us to test interactions with and associations within subgroups such as sex, chronological age, military status, and other variables. This large sample also constitutes a limitation, as combining data from many different sites and scanners may introduce confounds, particularly if there is a stratification of variables of interest by site/scanner. A second limitation is the use of a single imaging modality, as multi‐modal imaging inputs such diffusion imaging and resting‐state functional connectivity have been successfully employed in brain age estimation (Cole, 2020). Lastly, a number of factors, some of which covary with PTSD, that influence the aging process such as socioeconomic status, cardiometabolic function, diet, environmental pollutants, head trauma, and substance use, could not be accounted for in our study (Dowling et al., 2008; Fotenos et al., 2008; Griesbach et al., 2018; Joseph et al., 2009; Mehta et al., 2015). Future research is warranted to further understand how these factors may contribute to aging processes in the context of PTSD. Our results provide evidence of a link between PTSD diagnosis and accelerated brain aging processes, influenced by sex and chronological age. We offer a framework for exploring neurobiological markers of accelerated aging and potential underlying processes in PTSD.

## CONFLICT OF INTEREST

Dr. Krystal is a consultant for AbbVie, Inc., Amgen, Astellas Pharma Global Development, Inc., AstraZeneca Pharmaceuticals, Biomedisyn Corporation, Bristol‐Myers Squibb, Eli Lilly and Company, Euthymics Bioscience, Inc., Neurovance, Inc., FORUM Pharmaceuticals, Janssen Research & Development, Lundbeck Research USA, Novartis Pharma AG, Otsuka America Pharmaceutical, Inc., Sage Therapeutics, Inc., Sunovion Pharmaceuticals, Inc., and Takeda Industries. She is also on the Scientific Advisory Board for Lohocla Research Corporation, Mnemosyne Pharmaceuticals, Inc., Naurex, Inc., and Pfizer; she is a stockholder in Biohaven Pharmaceuticals, holds stock options in Mnemosyne Pharmaceuticals, Inc., holds patents for Dopamine and Noradrenergic Reuptake Inhibitors in Treatment of Schizophrenia, US Patent No. 5,447,948 (issued September 5, 1995), and Glutamate Modulating Agents in the Treatment of Mental Disorders, US Patent No. 8,778,979 (issued July 15, 2014) and filed a patent for Intranasal Administration of Ketamine to Treat Depression. US Application No. 14/197,767 (filed on March 5, 2014); US application or Patent Cooperation Treaty international application No. 14/306,382 (filed on June 17, 2014). She also filed a patent for using mTOR inhibitors to augment the effects of antidepressants (filed on August 20, 2018). Dr. Abdallah has served as a consultant, speaker and/or on advisory boards for FSV7, Lundbeck, Psilocybin Labs, Genentech and Janssen, and editor of Chronic Stress for Sage Publications, Inc.; he has filed a patent for using mTOR inhibitors to augment the effects of antidepressants (filed on August 20, 2018). Dr. Schmahl is consultant for Boehringer Ingelheim International GmbH. Dr. Davidson is the founder and president of, and serves on the board of directors for, the non‐profit organization Healthy Minds Innovations, Inc. Dr. Thompson received partial research support from Biogen, Inc. (Boston, USA) for research unrelated to the topic of this manuscript. Dr. Cole is a consultant to and shareholder in Brain Key, a medical image analysis software company.

### PEER REVIEW

The peer review history for this article is available at https://publons.com/publon/10.1002/brb3.2413

## Supporting information

Supporting Information

## Data Availability

The data that support the findings of this study are available from the corresponding author upon reasonable request.
