# Supplementary material for: Assessment of brain age in posttraumatic stress disorder: Findings from the ENIGMA PTSD and brain age working groups
Source: Brain Behav. 2021 Dec 14;12(1):e2413. doi: 10.1002/brb3.2413 (PMC8785613; doi:10.1002/brb3.2413)
Supplement: Supplementary file 1 — Supporting Information [file BRB3-12-e2413-s001.docx]

**Supplemental Information**

**Assessment of Brain Age in Posttraumatic Stress Disorder: Findings from the ENIGMA PTSD and Brain Age Working Groups**

**Corresponding Author**:

Ashley N. Clausen, PhD

Email: Ashley.n.clausen@gmail.com

**Method**

*Clinical Assessment*

**PTSD Severity and Diagnosis.** Current PTSD diagnosis was measured with one of the following instruments across all sites: Clinician Administered PTSD Scale (CAPS) for DSM-IV [1] or DSM-5 [2], the PTSD symptom checklist for DSM-IV Civilian (PCL-C) [3], Military (PCL-M) [4] versions, or for DSM 5 (PCL-5) [3], Davidson Trauma Scale (DTS; [5], the structured clinical interview for DSM-IV (SCID) [6] or DSM-5 [7] disorders, or the Mini International Neuropsychiatric Inventory (MINI) 6.0 [8] or 7.0 [9] . To ensure diagnosis was assessed consistently across sites, diagnostic cut scores were used to determine presence or absence of PTSD diagnosis whenever possible (see Table S1). The cut-off score for CAPS-IV and CAPS-5 was ≥ 40 and ≥ 23 [3, 7], respectively. For the PCL-C and PCL-M the cut score was ≥ 45, and ≥ 23 for the PCL-5 [3]. A cut score of ≥ 40 was used for the DTS [5]. The MINI and SCID were used to assess presence or absence of PTSD (categorical diagnosis), and thus no cut score was applied.

**Depression diagnosis and Severity**. Twenty of the 21 participating sites diagnosed depression and/or its severity with one of the following instruments: the Beck-Depression Inventory (BDI) – II [10] and 1A [11], the Hamilton Rating Scale for Depression (HAM-D) [12], the Hospital Anxiety and Depression Scale (HADS) [13], the Physical Health Questionnaire – 9 (PHQ-9) [14], the Center for Epidemiology Studies – Depression (CES-D) [15], the Depression and Anxiety Stress Scale (DASS) – depression subscale [16], the MINI, or the SCID. Cut-off scores were used to determine depression diagnosis. The cut-off score for the BDI was ≥ 20 (BDI-II) [10] or ≥ 15 (BDI-1A) [11], HAM-D was ≥ 20 [17], HADS was ≥ 11 [13], PHQ-9 was ≥ 10 [14], CES-D was ≥ 16 [15], and DASS was ≥ 21. The MINI and SCID were used to assess presence or absence of depression diagnosis and did not rely on cut-off scores.

*Machine-Learning Pipelines*

We compared three pre-trained machine-learning pipelines in a subset (N=386) of control subjects from three sites to assess the predictive accuracy of brain age estimates. Each pipeline is described below.

**BrainageR Pipeline**. The first step of the brainageR algorithm segments the T1 scans into gray matter and white matter, which are normalized to standard space using non-linear spatial registration. The two brain matter images are then concentrated and converted into a similarity matrix. Gaussian-process regression models are then used to predict chronological age [18]. The voxel-wise pipeline was previously trained on 2001 people without neurological disorders, psychiatric disorders, head trauma, and/or other medical illnesses. Brain age estimates from this pipeline using a cross-validation approach accounted for 88% of variance in chronological age with a MAE of 5.02 and a RMSE of 6.31 [19]. BrainageR is freely available for use (<https://github.com/james-cole/brainageR>; DOI: 10.5281/zenodo.3476365).

**ROI Pipelines**. We tested two pipelines that rely on FreeSurfer 5.3 autosegmentation of cortical and subcortical regions. The first, the PHOTON Brain Age pipeline (referred to hereafter as PHOTON-BA), was developed by the ENIGMA-MDD working group [20] using the PHOTON applications program interface (API). PHOTON was developed in Python as an API to design and optimize machine learning pipelines. The PHOTON-BA pipeline estimates brain age by applying a ridge regression model to subcortical volume of 14 regions including the nucleus accumbens, amygdala, caudate, hippocampus, pallidum, putamen, and thalamus, volume of the lateral ventricles, cortical thickness, and surface area of 68 cortical regions, as well as intracranial volume. The PHOTON-BA pipeline was trained in a sample of 1,147 adult males and females.

The second FreeSurfer-based pipeline that was tested was the Brain-Age Regression Analysis and Computation Utility Software (BARACUS v1.1.2; [21]). BARACUS uses linear support vector regression models to predict brain age derived from individuals’ FreeSurfer 5.3.0 cortical thickness, cortical surface area, and subcortical volumes. Specifically, native surface models for cortical thickness and surface area computed in FreeSurfer 5.3.0 were transformed into the fsaverage4 standard space, and subcortical segmentation metrics were extracted from the FreeSurfer aseg.stats file. We used the FILE mode docker on Ubuntu 16.04 using the Objective Cognitive Impairment (OCI) norm database (Liem2016_OCI_norm), which is trained on 1,166 individuals with no OCI (566 women / 600 men; mean age 59.1; SD = 15.2; age range 20-80 years) to produce the “stacked-anatomy” measure used for the predicted brain age.

**Results**

*Brain Age Prediction Performance*

We examined the predictive ability of the voxel-wise, PHOTON-BA, and BARACUS machine learning pipelines to estimate brain age relative to chronological age in a sub-sample of control subjects (n=386) from three sites. The voxel-based pipeline demonstrated a stronger relationship between brain age and chronological age (ICC=0.71, R=0.72 ; R^2^=0.51) and lower mean absolute error (MAE=5.68; RSME=7.46) compared to the PHOTON-BA (ICC=0.62; R=0.62; R^2^ =0.38; MAE=6.38; RMSE=8.10) and BARACUS (ICC=0.47; R=0.64; R^2^=0.41; MAE=8.8; RMSE=11.36) pipelines. Results of pipeline comparisons separated by site are presented in Figures S2 and S3.

*Impact of PTSD Diagnosses on Residual Estimates of Brain Age*

Residual brain age was calculated for each subject using the brainageR pipeline by regressing chronological age onto brain PAD [22]. Parallel linear mixed effects models were conducted to brain PAD were completed for residualized brainage.

Table S1: Measures for PTSD and Depression Assessment by Site

| Site Name  (N=total subjects) | | PTSD Diagnosis (n=included sample) | | PTSD Severity  (n=included sample) | | PTSD Severity Cutoff | Childhood Trauma  (n=included sample) | | MDD Diagnosis  (n=included sample) | | MDD Severity  (n=included sample) | | MDD Severity Cutoff |
| --- | --- | --- | --- | --- | --- | --- | --- | --- | --- | --- | --- | --- | --- |
| Amsterdam University Medical Centers, Location Academic Medical Center (N= 74) | | CAPS-4 (n = 74) | | CAPS-4 (n = 74) | | >=40 | ETI (n=74) | | HADS-D (n=74) | | HADS-D (n=72) | | >= 11 |
|  | |  | |  | |  |  | |  | |  | |  |
| Institute of Psychology, Chinese Academy of Sciences (N= 88) | | PCL-5 (n=88) | | PCL-5  (n=84) | | >=34 | N/A | | CES-D (n=83) | | CES-D (n=83) | | >=16 |
|  | |  | |  | |  |  | |  | |  | |  |
| Columbia University (N=84) | | CAPS-4 (n=84) | | CAPS-4  (n=84) | | >=40 | N/A | | HAMD (n=0) | | HAMD (n=0) | | >=20 |
|  | |  | |  | |  |  | |  | |  | |  |
| Minneapolis VA Health Care System (N=248) | | CAPS-4 (n=248) | | CAPS-4 (n=192) | | >=40 | N/A | | SCID (n=174) | | BDI (n=174) | | N/A |
|  |  | |  | |  | | |  | |  | |  |  |
| Duke University (N=206) | CAPS-5 (n=51)  CAPS-4(n=144)  SCID (n=10)  DTS (n=1) | | CAPS-5 (n=51)  CAPS-4 (n=144)  DTS (n=1)  N/A (n=10) | | >=23; >=40; N/A; >=40 | | | CTQ (n=156) | | BDI (n=163) | | BDI (n=163) | >=20 |
|  | |  | |  | |  |  | |  | |  | |  |
| Emory University: Grady Trauma Project (N=87) | | CAPS-4 (n=63)  CAPS-5 (n=24) | | CAPS-4 (n=63)  CAPS-5 (n=24) | | >=40;>=40;>=23 | CTQ (n=86) | | BDI (n=87) | | BDI (n=87) | | >=20 |
|  | |  | |  | |  |  | |  | |  | |  |
| Ghent University (Belgium) (N=67) | | MINI (n=67) | | N/A | | N/A | SLESQ-R (n=67) | | BDI (n=67) | | BDI (n=66) | | >=20 |
| University of Groningen, Groningen,  The Netherlands (N=41) | | CAPS-4 (n=41) | | CAPS-4 (n=41) | | >=40 | CTQ (n=41) | | BDI (n=41) | | BDI (n=38) | | >=20 |
|  | |  | |  | |  |  | |  | |  | |  |
| University of Wisconsin-Madison (N=58) | | CAPS-4 (n=58) | | CAPS-4 (n=58) | | >=40 | N/A | | BDI (n=0) | | BDI (n=58) | | >=20 |
|  | |  | |  | |  |  | |  | |  | |  |
| Injury and Traumatic Stress Clinical Consortium  (N=362) | | MINI (n=17) PCL-C (n=345) | | PCL-C (n=0)  PCL-C (n=345) | | >=45 | CTQ (n=362) | | PHQ-9 (n=0) | | PHQ-9 (n=356) | | >=10 |
|  | |  | |  | |  |  | |  | |  | |  |
| University of Wisconsin-Milwaukee: Milwaukee Trauma Outcomes Project (N=69) | | CAPS-5 (n=69) | | CAPS-5 (n=69) | | >=23 | CTQ (n=66) | | DASS Depression Subscale (n=66) | | DASS Depression Subscale (n=66) | | >=21 |
|  | |  | |  | |  |  | |  | |  | |  |
| Lawson Health Research Institute (Canada) (N=80) | | CAPS-4 (n=80) | | CAPS-4 (n=80) | | >=40 | CTQ (n=75) | | BDI (n=79) | | BDI (n=79) | | >=20 |
|  | |  | |  | |  |  | |  | |  | |  |
| Heidelberg University  (Germany) (N=49) | | SCID (n=49) | | DTS (n=49) | | >=40 | CTQ (n=49) | | BDI (n=49) | | BDI (n=49) | | >=20 |
|  | |  | |  | |  |  | |  | |  | |  |
| McLean Hospital (N=80) | | CAPS-5 (n=80) | | CAPS-5 (n=80) | | >=23 | CTQ (n=77) | | BDI-II (n=79) | | BDI-II (n=79) | | >=20 |
|  | |  | |  | |  |  | |  | |  | |  |
| Institute of Medical Psychology and Systems Neuroscience, University of Muenster (N=47) | | SCID (n=47) | | N/A | | N/A | N/A | | BDI (n=0) | | BDI (n=47) | | >=20 |
|  | |  | |  | |  |  | |  | |  | |  |
| VA Ann Arbor Health System and University of Michigan (N=46) | | CAPS-4 (n=46) | | PCL-M (n=36) | | >=45 | N/A | | BDI (n=26) | | BDI (n=26) | | >=20 |
|  | |  | |  | |  |  | |  | |  | |  |
| McLean Hospital  (N=112) | | CAPS-4 (n=112) | | CAPS-4 (n=73); N/A | | >=40 | CTQ (n=111) | | BDI-1A (n=112) | | BDI-1A (n=111) | | >=15 |
|  | |  | |  | |  |  | |  | |  | |  |
| University of South Dakota (N=122) | | PCL-M (n=94); PCL-C (n=28) | | PCL-M (n=94); PCL-C (n=28) | | >=45 | N/A | | CES-D (n=93); BDI (n=28) | | CES-D (n=93); BDI (n=28) | | >=16; >=20 |
|  | |  | |  | |  |  | |  | |  | |  |
| Stanford University (N=201) | | CAPS-4 (n=199) PCL-C (n=2) | | CAPS-4 (n=199)  PCL-C (n=2) | | >=40 ; >=45 | CTQ (n=146) | | N/A | | N/A | | N/A |
|  | |  | |  | |  |  | |  | |  | |  |
| Vanderbilt University (N=50) | | CAPS-5 (n=50) | | CAPS-5 (n=50) | | >=23 | CTQ (n=0) | | MINI (n=50) | | N/A | | N/A |
|  | |  | |  | |  |  | |  | |  | |  |
| West Haven Veterans Affairs (N=65) | | CAPS-4 (n=65) | | CAPS-4 (n=65) | | >=40 | N/A | | SCID (n=65) | | BDI (n=65) | | >=20 |
|  | |  | |  | |  |  | |  | |  | |  |

Notes: PTSD = posttraumatic stress disorder; N = total subjects; n = included sample; CAPS-4 = Clinician-Administered PTSD Scale, 4^th^ Edition; CAPS-5 = Clinician-Administered PTSD Scale, 5^th^ Edition; PCL-5 = posttraumatic stress disorder Checklist for DSM-5; PCL-C = posttraumatic stress disorder Checklist- Civilian Version; PCL-M = posttraumatic stress disorder Checklist- Military Version; MINI = The Mini International Neuropsychiatric Interview; SCID- Structural Clinical Interview for DSM Diagnoses; DTS = Davidson Trauma Scale; CTQ = Childhood Trauma Questionnaire; ETI = Early Trauma Inventory; SLESQ-R = Stressful Life Events Screening Questionnaire- Revised; HADS-D = Hospital Anxiety and Depression Scale- Depression; CES-D = Center for Epidemiological Studies-Depression Scale; HAMD = Hamilton Depression Rating Scale; BDI = Beck Depression Inventory; BDI 1A = Beck Depression Inventory Version 1A; BDI-II = Beck Depression Inventory Version II; PHQ = Personal Health Questionnaire; DASS Depression Subscale = Depression Anxiety Stress Scale.

Table S2. MRI Acquisition by Site

| Site Name | Manufacturer | Scanner Model | Head Coil Channels | Acquisition Sequence | Voxel size | FOV (mm) | Acquisition Orientation | TR / TE (milliseconds /  seconds) | Flip Angle |
| --- | --- | --- | --- | --- | --- | --- | --- | --- | --- |
| Amsterdam University Medical Centers, Location Academic Medical Center | Philips | Achieva | 32 | FAST MPRAGE | 1 x 1 x 1 | 240x188 | Axial | 8200 / 3.8 | 8 |
| Institute of Psychology, Chinese Academy of Sciences | Philips | Achieva | 8 | EPI | 1 x 1 x 1 | 220X220 | Axial | 8500 / 3.7 | 8 |
| Columbia University | GE | 1.5T SIGNA EXCITE | 8 | SPGR | 3.5 x 3.5 x 2.2 | 224X224 | Axial | 3000 /3.0 | 84 |
| Minneapolis VA Health Care System | Siemens | TIM Trio | 12 | MPRAGE | 1 x 1 x 1 | 256X256 | Coronal | 2530 / 3.7 | 7 |
| Duke University | GE | MR750 3T | 8 | FSPGR BRAVO | 0.9375 x 0.9375 x 0.9375 | 256x256 | Axial | 8160 / 3.2 | 12 |
|  | GE | EXCITE HD 3T | 8 | FSPGR BRAVO | 1 x 1 x 1 | 240x240 | Axial | 8148 / 3.2; 7840 / 2.9; 8160 / 3.2 | 12 |
|  | GE | 4T LX Nvi | 8 | Spin-echo co-planar | 1 x 1 x 1.9 | 240x240 | Axial | 12000 / 5.4 | 20 |
|  | Philips | Ingenia | 8 | 3D TFE SENSE | 0.9375 x 0.9375 x 1 | 240 x 240 | Axial | 8148 /3.7 | 8 |
| Emory University: Grady Trauma Project | Siemens | TIM Trio | 12 | MPRAGE | 1 x 1 x 1 | 224x256 | Axial | 2600 / 3 | 8 |
| Ghent University (Belgium) | Siemens | TIM Trio | 32 | MPRAGE | 1 x 1 x 1 | 256 x 256 | Transversal | 2250 / 4.18 | 9 |
| University of Groningen, Groningen, The Netherlands | Siemens | TIM Trio | 12 | MPRAGE | 1 x 1 x 1 | 256X256 | Sagittal | 1900 / 2.5 | 9 |
| University of Wisconsin-Madison | GE | X750 Discovery | 8 | BRAVO | 1 x 1 x 1 | 256x256 | Axial | 8.16 / 3.18 | 12 |
| Injury and Traumatic Stress Clinical Consortium | GE | Multiple | 8 | BRAVO | 1 x 1 x 1 | 256x256 | Sagittal | 9150 / 3.7 | 10 |
|  | Siemens | Multiple | 12 | MPRAGE | 1 x 1 x 1 | 256x256 | Sagittal | 2530 / 3.32 | 7 |
|  | Philips | Multiple | 8 | 3D TFE  SENSE | 1 x 1 x 1 | 256x256 | Sagittal | 7600 / 3.5 | 7 |
| University of Wisconsin-Milwaukee: Milwaukee Trauma Outcomes Project | GE | MR750 Signa Excite | 32 | SPGR | 1 x 0.9375 x 0.9375 | 240 | Sagittal | 8.2 / 3.2 | 12 |
| Lawson Health Research Institute | Siemens | TIM Trio | 32 | MPRAGE | 1 x 1 x 1 | 256x240 | Sagittal | 2300 / 2.98 | 9 |
| Heidelberg University | Siemens | TIM Trio | 32 | SPGR | 1 x 1 x 1 | 192x192 | Axial | 2000 / 3 | 80 |
| McLean Hospital | Siemens | TIM Trio | 12 | MEMPRAGE TFL | 1 x 1 x 1 | 256 | Sagittal | 2530 / 1.64, 3.5, 5.36, 7.22 | 7 |
| Institute of Medical Psychology and Systems Neuroscience, University of Muenster | Siemens | Magnetom Prisma | 32 | MPRAGE | 1 x 1 x 1 | 256 | Sagittal | 2130 / 2.28 | 8 |
| VA Ann Arbor Health System and University of Michigan | GE | Signa | 8 | Gradient-echo spiral pulse | 1 x 1 x 1 | 240x240 | - | 25 / 6.6 | 90 |
| McLean Hospital | Siemens | TIM Trio | 32 | MEMPRAGE | 1.3 X 1 X 1.3 | 256X128 | Sagittal | 2530 / 3.31 | 7 |
| University of South Dakota | Siemens | Skyra | 20 | MPRAGE | 1 x 1 x 1 | 240x240x180; 256x256x256 | Sagittal | 1900 / 2.13 | 9 |
| Stanford University | GE | MR750 3T | 8 | 3D SPGR | 1.5 x 0.9 x 1.1 | 220X220; 240x240 | Coronal | 8 / 3.6; 8.6 / 3.4 | 15 |
| Vanderbilt University |  |  |  |  |  |  |  |  |  |
| West Haven Veterans Affairs | Siemens | TIM Trio | 12 | MPRAGE | 1 x 1 x 1 | 256x256 | Sagittal | 2530 / 2.71 | 7 |

Notes: FOV = field of view; TR = repetition time; TE = echo time; GE = General Electric; MPRAGE = magnetization-prepared rapid gradient echo; SPGR = spoiled gradient recall; MEMPRAGE = multi-echo magnetization-prepared rapid gradient echo ; BRAVO = inversion preparation of a fast low-angle spoiled gradient recall; TFE SENSE = turbo field echo sensitivity encoding; EPI = echo planar image ; FAST = fast low-angle shot; TFL = turbo flash.

| Table S3. Linear Mixed Effects (LME) Results for PTSD Diagnosis and Residualized Predicted Brain Age Difference | | | | | |  | |
| --- | --- | --- | --- | --- | --- | --- | --- |
|  |  | **Beta** | **CI** | **t-value** | ***p*** | |  |
| ***Model 1*** *AIC = 15335.4, BIC = 15392.5, N = 2229, Marginal R^2^ / Conditional R^2^ = 0.220 / 0.266* | | | | | | | |
|  | Intercept | -1.13 | -2.18 – -0.08 | -2.12 | 0.040 | |  |
|  | PTSD | 0.28 | -0.40 – 0.96 | 0.81 | 0.420 | |  |
|  | Age | -0.38 | -0.41 – -0.34 | -22.38 | <0.001* | |  |
|  | Age^2^ | 0.00 | -0.00 – 0.00 | 0.644 | 0.520 | |  |
|  | Sex (M) | 0.84 | 0.10 – 1.58 | 2.21 | 0.027* | |  |
|  |  |  |  |  |  | |  |
| **** ***Model 2*** *AIC = 15336.9, BIC = 15399.7, N = 2229, Marginal R^2^ / Conditional R^2^ = 0.226 / 0.270* | | | | | | | |
|  | Intercept | -1.36 | -2.45 – -0.28 | -2.46 | 0.017 | |  |
|  | PTSD | 0.75 | -0.29 – 1.80 | 1.43 | 0.153 | |  |
|  | Age | -0.40 | -0.45 – -0.34 | -13.94 | <0.001* | |  |
|  | Age^2^ | 0.00 | -0.00 – 0.00 | 0.69 | 0.493 | |  |
|  | Sex (M) | 1.15 | 0.26 – 2.04 | 2.53 | 0.017* | |  |
|  | PTSD*Age | -0.00 | -0.09 – 0.08 | -0.14 | 0.890 | |  |
|  | PTSD*Sex (M) | -0.76 | -2.09 – 0.57 | -1.11 | 0.265 | |  |
|  | Age*Sex (M) | 0.09 | 0.02 – 0.16 | 2.39 | 0.017* | |  |
|  | PTSD*Age*Sex (M) | -0.14 | -0.26 – -0.02 | -2.34 | 0.019* | |  |
|  |  |  |  |  |  | |  |
| ***Model 3*** *AIC = 15338.7, BIC = 15407.3, N = 2229, Marginal R^2^ / Conditional R^2^ = 0.227 / 0.270* | | | | | | | |
|  | Intercept | -1.23 | -2.46 – -0.01 | -1.97 | 0.053 | |  |
|  | PTSD | 0.76 | -0.28 – 1.80 | 1.43 | 0.150 | |  |
|  | Age | -0.40 | -0.45 – -0.34 | -13.93 | <0.001* | |  |
|  | Age^2^ | 0.00 | -0.00 – 0.00 | 0.69 | 0.488 | |  |
|  | Sex (M) | 1.14 | 0.25 – 2.04 | 2.50 | 0.012* | |  |
|  | Race | -0.03 | -0.18 – 0.11 | -0.44 | 0.664 | |  |
|  | PTSD*Age | -0.01 | -0.09 – 0.08 | -0.15 | 0.883 | |  |
|  | PTSD*Sex (M) | -0.76 | -2.09 – 0.58 | -1.11 | 0.267 | |  |
|  | Age*Sex (M) | 0.09 | 0.02 – 0.16 | 2.38 | 0.018* | |  |
|  | PTSD*Age*Sex (M) | -0.14 | -0.26 – -0.02 | -2.33 | 0.020* | |  |
|  |  |  |  |  |  | |  |
| ***Model 4*** *AIC = 14472.6, BIC = 14540.4, N = 2102, Marginal R^2^ / Conditional R^2^ = 0.247 / 0.292* | | | | | | | |
|  | Intercept | -1.75 | -2.93 – -0.57 | -2.91 | 0.005 | |  |
|  | PTSD | 0.79 | -0.26 – 1.85 | 1.48 | 0.140 | |  |
|  | Age | -0.42 | -0.48 – -0.37 | -14.00 | <0.001* | |  |
|  | Age^2^ | 0.00 | -0.00 – 0.00 | 0.64 | 0.524 | |  |
|  | Sex (M) | 1.11 | 0.14 – 2.08 | 2.25 | 0.025 | |  |
|  | Military Status | 1.06 | -0.25 – 2.36 | 1.59 | 0.114 | |  |
|  | PTSD*Age | 0.02 | -0.07 – 0.10 | 0.40 | 0.689 | |  |
|  | PTSD*Sex (M) | -0.88 | -2.25 – 0.48 | -1.27 | 0.206 | |  |
|  | Age*Sex (M) | 0.09 | 0.01 – 0.16 | 2.17 | 0.030 | |  |
|  | PTSD*Age*Sex (M) | -0.15 | -0.27 – -0.03 | -2.4 | 0.017 | |  |

| Table S4. Linear Mixed Effects (LME) Results for PTSD Severity | | | | |  |  |
| --- | --- | --- | --- | --- | --- | --- |
|  |  | Beta | CI | t-value | p |  |
| *LME 1 AIC = 3551.2, BIC = 3576.7, N = 515, Marginal R^2^ / Conditional R^2^ = 0.266/0.291* | | | | | | |
|  | Intercept | 1.07 | -1.85 – 3.98 | 0.72 | 0.474 |  |
|  | PTSD Severity | -0.01 | -0.05 – 0.03 | -0.67 | 0.504 |  |
|  | Age | -0.47 | -0.54 – -0.40 | -12.44 | <0.001 |  |
|  | Age^2^ | 0.004 | -0.00 – 0.01 | 1.33 | 0.183 |  |
|  |  |  |  |  |  |  |
| *LME 2 AIC = 3550.9, BIC = 3580.6, N = 515, Marginal R^2^ / Conditional R^2^ = 0.269 / 0.293* | | | | | | |
|  | Intercept | 1.02 | -1.88 – 3.92 | 0.96 | 0.491 |  |
|  | PTSD Severity | -0.01 | -0.05 – 0.03 | -0.64 | 0.524 |  |
|  | Age | -0.66 | -0.92 – -0.40 | -5.03 | <0.001 |  |
|  | Age^2^ | 0.00 | -0.00 – 0.01 | 1.22 | 0.224 |  |
|  | PTSD Severity * Age | 0.003 | -0.00 – 0.01 | 1.54 | 0.125 |  |

Note: PTSD = posttraumatic stress disorder; LME = linear mixed effects; AIC = Akaike’s Information Criteria; BIC = Bayesian Information Criteria


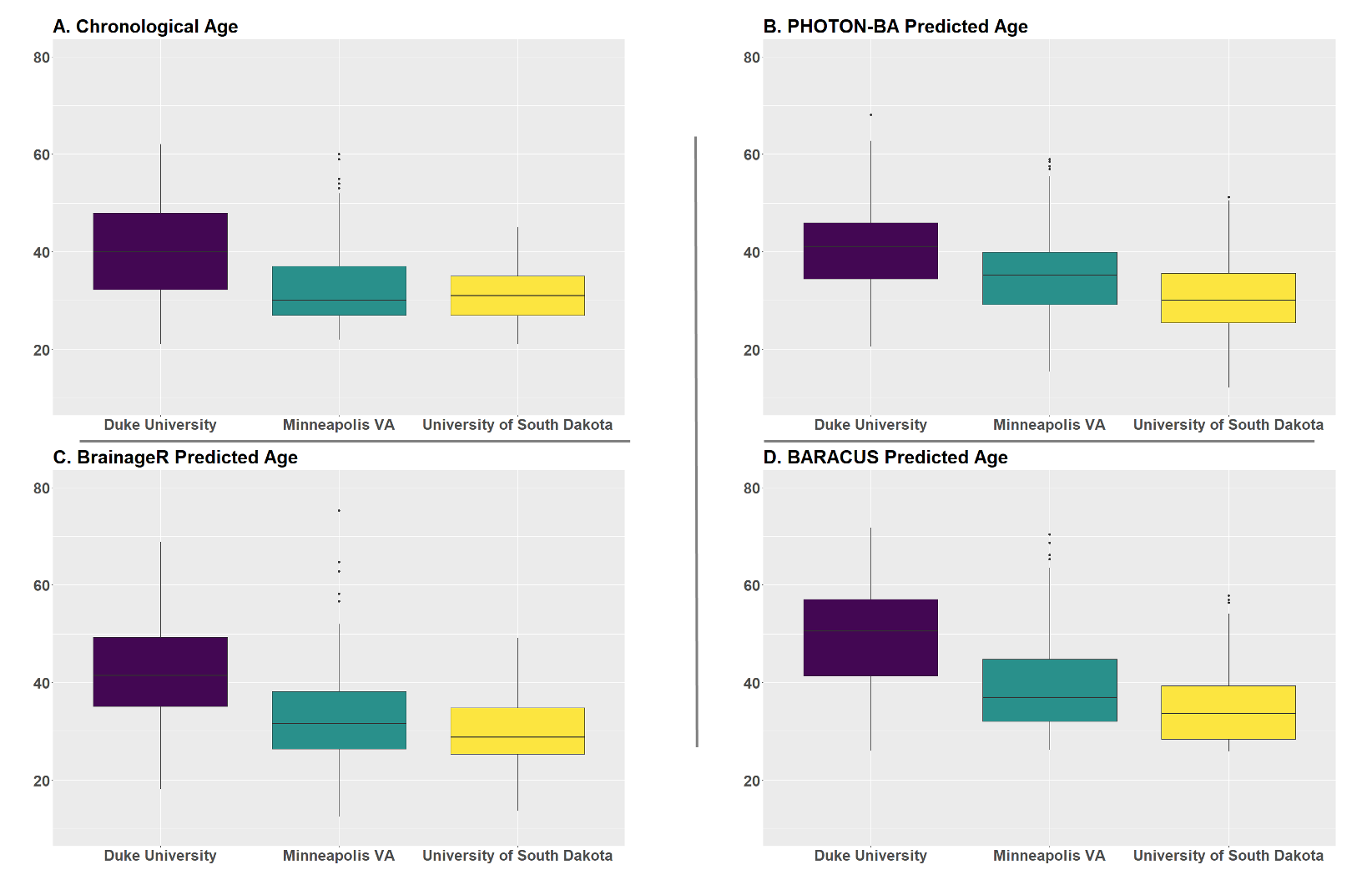


**Figure S1**. Chronological Age and Predicted Brain Age by Site. This figure depicts A) chronological age, B), estimated brain age using the PHOTON-brain age (BA) method, C) estimated brain age using the voxel-based method (BrainageR), and D) estimated brain age using the Brain-Age Regression Analysis and Computation Utility Software (BARACUS) method. A similar pattern emerged across all ages, in that, subjects from Duke University displayed higher chronological age and predicted brain age across all methods relative to subjects from the Minneapolis VA Health Care System and University of South Dakota. Thus, supplemental analyses were conducted by site and collapsed across sites.


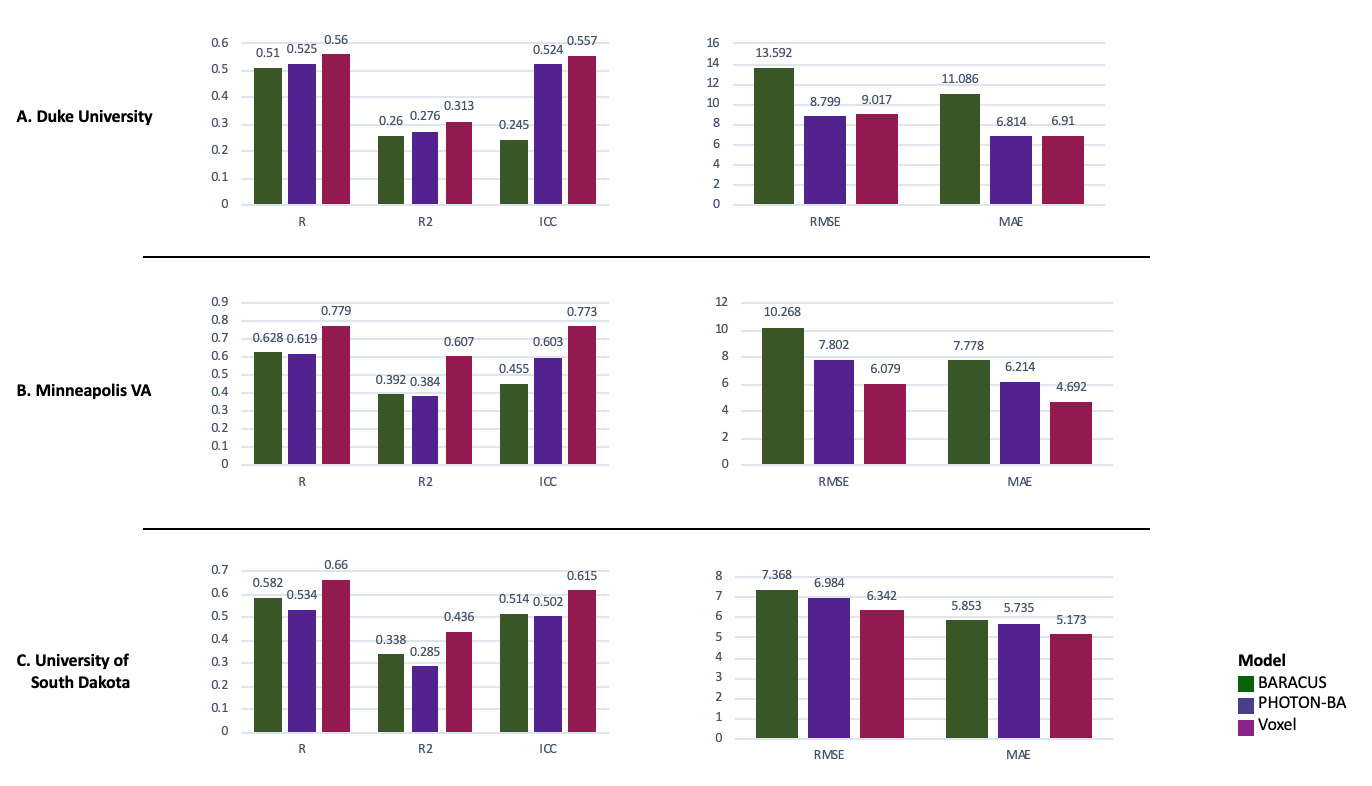


**Figure S2**. Performance metrics of each brain age estimation model including R, R^2^(R2), intra-class correlation (ICC), root mean square error (RMSE) and median absolute error (MAE), by site. The Brain-Age Regression Analysis and Computation Utility Software (BARACUS) model is represented by the green bars, the PHOTON-Brain Age (BA) model is represented by the purple bars, and the Voxel model is represented by the maroon bars. Metrics are presented by site: A) Duke University, B) Minneapolis VA Health Care System, and C) University of South Dakota. Across sites, the Voxel method displayed the strongest relationship between chronological and predicted brain age (R = .56 – 0.779; R^2^ = 0.31 – 0.61), with moderate reliability (ICC = 0.56 – 0.77). While subjects in the Duke University sample showed slightly lower error (RMSE and MAE in) using the PHOTON-BA method versus the Voxel, Minneapolis VA and University of South Dakota showed lower error for the Voxel method. Across all sites, the BARACUS model showed the highest error.


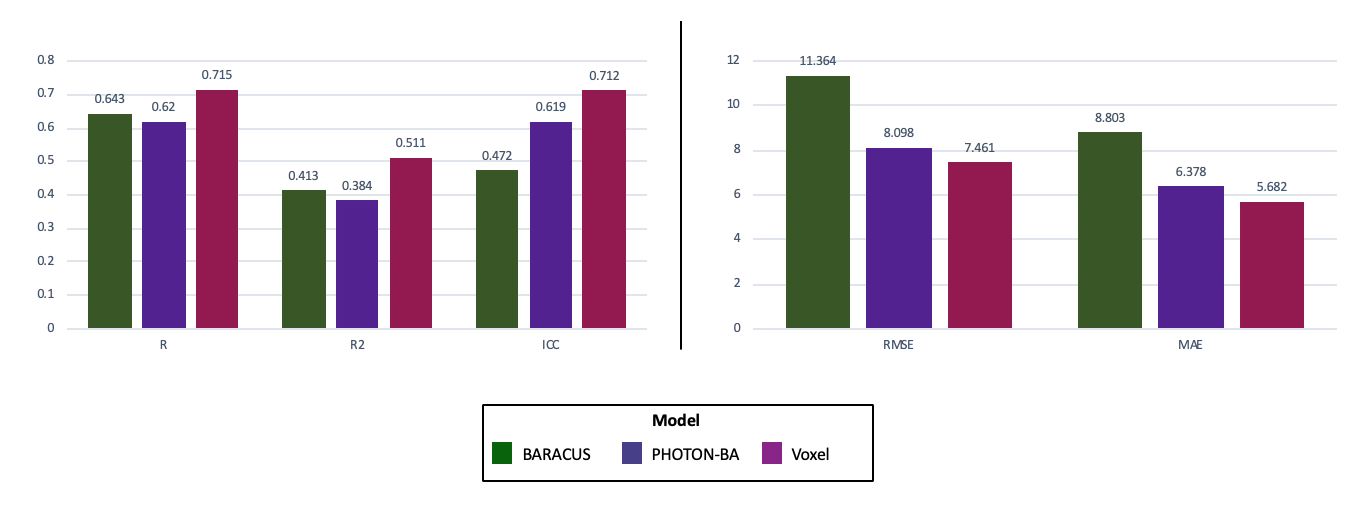


**Figure S3**. Performance metrics of each brain age estimation model including R, R^2^ (R2), intra-class correlation (ICC), root mean square error (RMSE) and median absolute error (MAE), collapsed across sites. The Brain-Age Regression Analysis and Computation Utility Software (BARACUS) model is represented by the green bars, the PHOTON-Brain Age (BA) model is represented by the purple bars, and the Voxel model is represented by the maroon bars. Across sites, the Voxel model showed the strongest relationship between chronological age and predicted brain age (R = 0.715, R^2^ = 0.511) relative to PHOTON-BA (R = 0.620, R^2^ = 0.384) and BARACUS (R = 0.643, R^2^ = 0.413) models. The Voxel model also showed the strongest reliability in predicting brain age (ICC = 0.712) compared to the PHOTON-BA (ICC = 0.619) and BARACUS (ICC = 0.472) models. Similarly, the Voxel method demonstrated the lowest error (RMSE = 7.461 years; MAE = 5.682 years) relative to the PHOTON-BA (RMSE = 8.098 years; MAE = 6.378 years) and BARACUS (RMSE = 13.364 years; MAE = 8.803 years) models.

**References**

1. Pfohl, B., N. Blum, and M. Zimmerman, *Structured interview for DSM-IV personality: Sidp-IV*. 1997: American Psychiatric Pub.

2. Weathers, F.W., et al., *The Clinician-Administered PTSD Scale for DSM–5 (CAPS-5): Development and initial psychometric evaluation in military veterans.* Psychological Assessment, 2018. **30**(3): p. 383.

3. Weathers, F.W., et al., *The ptsd checklist for dsm-5 (pcl-5).* Scale available from the National Center for PTSD at www. ptsd. va. gov, 2013. **10**.

4. Yarvis, J.S., et al., *Assessment of PTSD in older veterans: the posttraumatic stress disorder checklist: military version (PCL-M).* Advances in Social Work, 2012. **13**(1): p. 185-202.

5. Davidson, J.R. *Davidson trauma scale (DTS)*. 1996. MHS.

6. Spitzer, R.L., et al., *The structured clinical interview for DSM-III-R (SCID): I: history, rationale, and description.* Archives of general psychiatry, 1992. **49**(8): p. 624-629.

7. First, M., et al., *Structured clinical interview for DSM-5 disorders–research version (SCID-5-RV).* Arlington: American Psychiatric Assocation, 2014.

8. Sheehan, D. and Y. Lecrubier, *The mini international neuropsychiatric interview version 6.0 (MINI 6.0).* Medical Outcomes System Inc.: Jacksonville, FL, 2010.

9. Sheehan, D.V., J.M. Giddens, and I.S. Sheehan, *Status update on the Sheehan-suicidality tracking scale (S-STS) 2014.* Innovations in clinical neuroscience, 2014. **11**(9-10): p. 93.

10. Beck, A.T., R.A. Steer, and G.K. Brown, *Beck depression inventory-II.* San Antonio, 1996. **78**(2): p. 490-498.

11. Steer, R.A., et al., *Common and specific dimensions of self-reported anxiety and depression: the BDI-II versus the BDI-IA.* Behaviour research and therapy, 1999. **37**(2): p. 183-190.

12. Bremner, J.D., E. Vermetten, and C.M. Mazure, *Development and preliminary psychometric properties of an instrument for the measurement of childhood trauma: the Early Trauma Inventory.* Depression and anxiety, 2000. **12**(1): p. 1-12.

13. Snaith, R. and A. Zigmond, *Hospital anxiety and depression scale (HADS).* Handbook of psychiatric measures. Washington, DC: American Psychiatric Association, 2000: p. 547-548.

14. Kroenke, K. and R.L. Spitzer, *The PHQ-9: a new depression diagnostic and severity measure.* Psychiatric annals, 2002. **32**(9): p. 509-515.

15. Orme, J.G., J. Reis, and E.J. Herz, *Factorial and discriminant validity of the center for epidemiological studies depression (CES‐D) scale.* Journal of clinical psychology, 1986. **42**(1): p. 28-33.

16. Lovibond, P.F. and S.H. Lovibond, *The structure of negative emotional states: Comparison of the Depression Anxiety Stress Scales (DASS) with the Beck Depression and Anxiety Inventories.* Behaviour research and therapy, 1995. **33**(3): p. 335-343.

17. Iannuzzo, R., et al. *The HAM-D/MADRS interview: An integrated depression symptom rating interview*. in *BIOLOGICAL PSYCHIATRY*. 2004. ELSEVIER SCIENCE INC 360 PARK AVE SOUTH, NEW YORK, NY 10010-1710 USA.

18. Cole, J.H., et al., *Brain age predicts mortality.* Molecular psychiatry, 2018. **23**(5): p. 1385-1392.

19. Cole, J.H., et al., *Predicting brain age with deep learning from raw imaging data results in a reliable and heritable biomarker.* NeuroImage, 2017. **163**: p. 115-124.

20. Schmaal, L., et al., *Neuroimaging, genetics, and personalized psychiatry: Developments and opportunities from the ENIGMA consortium*, in *Personalized Psychiatry*. 2020, Elsevier. p. 483-497.

21. Liem, F., et al., *Predicting brain-age from multimodal imaging data captures cognitive impairment.* Neuroimage, 2017. **148**: p. 179-188.

22. Le, T.T., et al., *A nonlinear simulation framework supports adjusting for age when analyzing BrainAGE.* Frontiers in aging neuroscience, 2018. **10**: p. 317.
